# Deep-EEG: An Optimized and Robust Framework and Method for EEG-Based Diagnosis of Epileptic Seizure

**DOI:** 10.3390/diagnostics13040773

**Published:** 2023-02-17

**Authors:** Waseem Ahmad Mir, Mohd Anjum, Izharuddin, Sana Shahab

**Affiliations:** 1 Department of Computer Engineering, Aligarh Muslim University, Aligarh 202002, India; 2 Department of Business Administration, College of Business Administration, Princess Nourah Bint Abdulrahman University, P.O. Box 84428, Riyadh 11671, Saudi Arabia

**Keywords:** epilepsy, seizure, deep learning, diagnosis, electroencephalogram, Bi-LSTM

## Abstract

Detecting brain disorders using deep learning methods has received much hype during the last few years. Increased depth leads to more computational efficiency, accuracy, and optimization and less loss. Epilepsy is one of the most common chronic neurological disorders characterized by repeated seizures. We have developed a deep learning model using Deep convolutional Autoencoder—Bidirectional Long Short Memory for Epileptic Seizure Detection (DCAE-ESD-Bi-LSTM) for automatic detection of seizures using EEG data. The significant feature of our model is that it has contributed to the accurate and optimized diagnosis of epilepsy in ideal and real-life situations. The results on the benchmark (CHB-MIT) dataset and the dataset collected by the authors show the relevance of the proposed approach over the baseline deep learning techniques by achieving an accuracy of 99.8%, classification accuracy of 99.7%, sensitivity of 99.8%, specificity and precision of 99.9% and F1 score of 99.6%. Our approach can contribute to the accurate and optimized detection of seizures while scaling the design rules and increasing performance without changing the network’s depth.

## 1. Introduction

Detection of brain disorders using deep learning methods has received much hype during the last few years [1]. Increased depth leads to more computational efficiency, making it possible to build high-performing, optimized, computationally efficient deep neural networks, stacking more hidden layers while maintaining the performance at par with state-of-the-art deep neural networks [2,3]. Epilepsy is a common and persistent neurological condition marked by frequent seizures [4]. Epilepsy affects an estimated 70 million individuals globally. The affected people span all age groups. After migraine, it is the second most prevalent neurological condition [5]. Epilepsy was defined conceptually in 2005 as a disorder of the brain characterized by an enduring predisposition to generate epileptic seizures that are not caused by established and treatable medical illnesses.

Seizures can have a variety of causes. They could be caused by a brain injury or a genetic predisposition, but in most cases, the causes are unknown [6]. Electroencephalography, which measures potentials in the brain region, can be used to record the electrical activities of the brain. A widely popular approach to detecting and diagnosing the epileptic disorder is to visually inspect the electroencephalogram (a prime signal for measuring the electrical activity of the brain) [7]. However, visually inspecting the EEG by medical professionals is a very labor-sensitive and time-consuming approach. Researchers have made several successful attempts to automate the diagnosis. These approaches are based on machine learning approaches employing feature extraction methods in the time and frequency domains [8,9].

Recent advances in deep learning have revolutionized the field of Artificial Intelligence and healthcare, particularly in disease diagnosis, providing promising results, which have led to state-of-the-art brain–computer interfaces. The term deep here refers to the linear sequence of layers, considered essential for the system’s high performance [10,11]. The success of deep networks is attributed to the increased representative ability of the network, which makes it learn abstract features [12].

We have devised a deep learning model based on Deep Convoluted Autoencoder Epileptic Seizure Detection Bidirectional-Long Short Memory (DCAE-ESD-Bi-LSTM) for epileptic seizure detection. Our model can aid in precisely and efficiently detecting seizures while scaling the design guidelines for improved performance. Our model’s key characteristic is that it accurately and optimally identifies seizures in ideal and realistic settings with little latency and computational cost. In this study, we have created the best deep network architecture based on bidirectional-long short-term memory for epilepsy detection by teaching temporal dependencies in time series EEG data. The most crucial features related to epileptic episodes are extracted using a fully linked layer. The anticipated labels are output using a SoftMax layer. Our model obtained 99.8% classification accuracy, 99.7% sensitivity, and 99.8% specificity on the benchmark dataset and a dataset amassed by the authors, the results demonstrate the higher importance of the suggested strategy over the baseline deep learning techniques. The model has also been shown to be reliable in challenging real-world scenarios.

Our method can aid in the precise and effective detection of seizures while scaling the rules for improved performance and design without altering the depth of the network. The deep method aids in the precise and optimal identification of seizures under both ideal and realistic circumstances.

## 2. Electroencephalogram (EEG)

**Figure 1:**
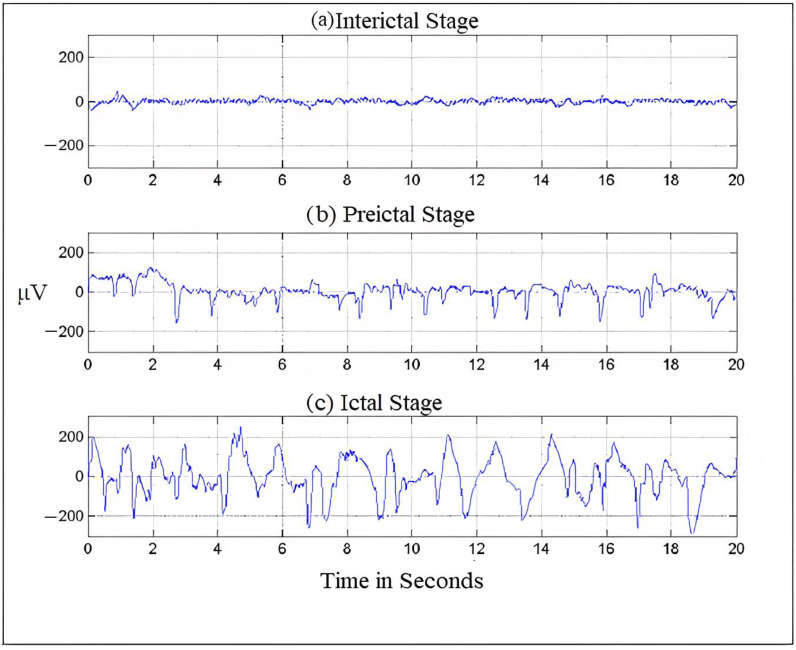
Brain- activity is recorded during different seizure stages.

Electroencephalography, which measures potentials in the brain region, can be used to record the electrical activities of the brain [13]. Figure 1 shows an EEG signal. The frequency of EEG signals is largely divided into five categories, typically known as frequency bands. The frequency distribution ranges from 0 to 100 Hz. The lowest band is the delta (δ), 0.5–4 Hz; other bands are as follows: theta (θ), 4–8 Hz; alpha (α), 8–13 Hz; beta (β), 13–30 Hz and finally, gamma (γ), which ranges from 30 Hz onwards. Both invasive and non-invasive methods can be used to register an electroencephalogram (EEG) [14]. The electrical potential of the scalp is monitored in the non-invasive mode, and then following surgery, nodes are planted in the intracranial region in the invasive mode [15]. We can diagnose brain problems like epilepsy, encephalopathy, or sleep disorders using the easily accessible EEG testing technology, which medical professionals widely use to analyze brain behavior. The EEG recordings are read by neurologists. Since the number of epileptic patients is increasing and there are very few neurologists available (only 1200 in India who are registered with the Indian Academy of Neurology) [16], the EEG segments are divided into five approximate and detailed sub-bands [17]. Then, employing wavelet coefficients in the low-frequency range of 0–32 Hz, the EEG’s energy and normalized coefficient features are determined. A linear discriminant analysis (LDA) classifier is used to show the potential of the recovered attributes in seizure onset detection.

### 2.1. Characteristic Nature of EEG Signals

Usually, frequency serves as the most important criterion for understanding the structural and behavioral functioning of the brain [18]. This understanding helps in assessing the abnormalities in the brain and cognitive research. Frequency is the rhythmic repetition of brain signals measured in cycles per second or Hertz (Hz) [19]. These brain waves are unpredictable and vary with age, state of sleep, or awakening. The frequency of EEG signals is largely divided into five categories, typically known as frequency bands. The frequency distribution ranges from 0 to 100 Hz. The lowest band is the delta (δ), 0.5–4 Hz; other bands are theta (θ), 4–8 Hz; alpha (α), 8–13 Hz; beta (β), 13–30 Hz and finally, gamma (γ), which ranges from 30 Hz onwards [4,20]. Figure 1 shows the seizure activity during the preictal, ictal, and interictal stages.

### 2.2. EEG Signal Analysis and Classification

Since data in the data repositories, both online and offline, have a large volume comprising various categories as the recordings are done over a long period, deep learning methods are used to analyze and classify this data through proper techniques to extract meaningful information from this large amount of data. EEG evaluation is usually performed by experienced professionals who manually visualize the EEG recordings [21,22]. However, the manual inspection of the signals has no standards that are set. Besides, it is very time-consuming and eventually results in errors. An automated system that could classify the EEG signals into normal and abnormal will help healthcare professionals and reduce human errors to a large extent. For classification purposes, EEG signals need to be carefully analyzed for accurate insight and a better understanding of the signals. Classifying time-varying non-stationary electroencephalogram signals is a challenge in neuroscience. Several classification methods using deep learning have been identified in the literature to classify different brain disorders using typical EEG patterns. The significance of brain disease diagnosis using artificial intelligence in healthcare is far from fully understood. However, the potential of deep learning in brain disorder diagnosis cannot be neglected due to the pace at which deep learning is employed in other health domains.

### 2.3. EEG Data Processing

The measurement of EEG signals to understand the workings of the brain is very critical, as human interventions contaminate the EEG signal, which is being measured using electrodes and subsequently digitized. The processing of EEG data is very important. Therefore, a systematic procedure is followed to filter the unwanted signals from the raw EEG signals. The steps followed are

1. Pre-processing: Raw EEG is preprocessed to improve signal quality without loss of information. The raw EEG signals are first denoised by removing the artifacts using filters to make clean and relevant information available [23].
2. Feature extraction: Brain disorders are characterized by certain patterns different from normal EEG signals. Therefore, feature extraction helps us describe the signals by the most relevant values, known as features [24].
3. Classification: Classification, also known as feature translation, classifies the feature sets extracted from the signals into different classes representing normal or pathological conditions.

### 2.4. Artifacts in EEG

Biomedical signals are usually weak in amplitude and power; therefore, they are susceptible to interferences and can be distorted in the presence of other signals. EEG recordings are also weak in amplitude; therefore, they are corrupted by several types of artifacts. These artifacts do not carry any information but affect the seizure patterns while degrading the process of signal processing [25]. Physiological processes, external environment, and instrumental noises contribute to EEG artifacts. Some of the artifacts are discussed here.

- Electromyogram: The skeletal muscle movements represented by electrical signals are known as electromyogram signals. During the acquisition of EEG signals, these signals interfere with the brain signals, causing contamination of the EEG data. EMGs have a high amplitude and a broad spectrum; even weak EMGs can cause interference in EEG recordings. Given the vulnerability of EEG signals to be contaminated by EMGs, it is very important to develop EMG correction tools [26]. During the preprocessing, these signals can be filtered using a 20–60-Hz Band Pass Filter.
- Eye movements: Eye movements and blinking cause interference in EEG signals. They distort the EEG signals, making the diagnosis of epileptic seizure a difficult job. They also reduce the signal-to-noise ratio (SNR) of EEG signals, thereby making the diagnosis of epilepsy more challenging [27]. Various methods have been proposed to correct the effects of eye movements. One is discarding the data corresponding to eye movements, and the other is filtering out the effect of ocular activity. This can be done by filtering the signals of eye movements through a Bandpass filter.
- White noise: There are other sources of interference that can be added up to and called by a common name, i.e., white noise. White noise includes instrumental noise, atmospheric noise, powerline interferences, and electrode resistance. These interferences are additive and generally have a Gaussian distribution.

## 3. Related Work

To automate the diagnosis of epilepsy, a variety of machine learning and deep learning techniques have been used. The following are some examples of related works. In their study, Arabi et al. [28] combined various EEG patterns recorded in the time, frequency, and time–frequency domains. The categorization of seizure and non-seizure was 93% accurate when the data was fed into a backpropagation neural network (BNN) classifier with two hidden layers fed with features and the EEG cepstral data. Syed Muhammad Usman et al [29] suggested a deep learning-based ensemble learning method for epileptic seizure prediction. According to the researchers, accurate prediction of epileptic seizures with a low false positive rate is still a challenge. Catarina da Silva Lourenco et al [30] collected EEGs for focal epilepsy, of 50 patients, for generalized epilepsy. They collected data from 49 patients and 67 controls were used on filtered data, subsampled, and divided into two-second periods. Then filtered data was used for automatic recognition of interictal epileptic discharges (IEDs) in EEG recordings which can decrease the time exhausted on visual analysis for the diagnosis of epilepsy. Data was augmented by incrementing the number of input samples including IEDs by temporal moving and making use of different montages. For the detection of IEDs, VGG C convolutional neural network was trained. This method decreased the false positive rate from 2.11 to 0.73 detection per minute without affecting sensitivity and specificity. A linear discriminant analysis (LDA) classifier was used to show the potential of the recovered attributes in seizure onset detection. The LDA classifier achieved a classification accuracy of 91.80%. Md.Rashed-Al-Mahefuz et al [31] focused on drawing and estimating deep convolutional neural network-based classifiers for seizure detection. Time domain signals were converted to the frequency domain. The proposed model achieved the highest average classification accuracy of 99.21% using the FT-VGG16 classifier. Jianjun Huang et al [32] presented recognition of epileptic foci in the local brain region that helps in inferring that there is a lesion through the classification result. The authors enlisted 59 children with hippocampus epilepsy and fed 70 more and diffusion kurtosis images (DKI) of subjects that were collected DKI repository. These images clarified the pathological modification of local tissues and any other regions of epileptic foci placed at the molecular level. A convolutional neural network (CNN) mounted on transfer learning techniques is designed for feature selection of FA, MD, MK, and the fusion of FA and MK with a support vector machine for the classification of epilepsy and normal control. The classifier has been able to produce an accuracy of 90.8%. Amin and Kamboh [33] used the CHB-MIT dataset to conduct patient-specific tests using the decision tree classifier and the RUS Boost algorithm to process imbalanced seizure/non-seizure data. They achieved an accuracy of 97%, and their model performed well while reducing the false positive rate and the system was able to learn quickly. By using a conventional neural network (CNN) besides channel minimization, Ranjan et al [34] represent an efficient seizure prediction technique. For the extraction of automatic features and the classification of epilepsy, the CNN model is used, which has been able to produce a classification accuracy of 99.47%. Through optimization the no of EEG channels has been reduced to 6 from 22 i.e., 72.73% deduction of channels, the method obtains sensitivity and specificity of 97.83% and 92.36% respectively. It achieves a 76.4% of the false positive rate. Truong et al. [18] developed an automatic seizure identification approach using intracranial electroencephalography (iEEG) data using supervised classifiers. They retrieved spectral power and correlations between channels as features in frequency and time domains. The classification was done using the Random Forest Classifier. Kiranyaz et al. [35] developed a model for seizure detection that achieved an average sensitivity of 89.01% and an average specificity of 94.71% on the CHB-MIT dataset. The approach’s computational complexity increased due to the high number of classifiers used. However, the model is patient-specific. Therefore, generalizability is limited. Fergus et al. [36] developed a method for seizure identification across participants based on conventional machine-learning approaches. By choosing features in various brain regions, they achieved 88% sensitivity and 88% specificity over the CHB-MIT dataset. Data filtering, feature extraction, feature selection, and training classifiers comprise the method’s primary four components. In cross-validation studies, EEG signals from CHB-MIT were divided into segments with a segment length of 60 s; one seizure segment was shortened for each seizure, and non-seizure segments were recovered from non-seizure EEG recordings in an equal number of seizure segments. The experiment was carried out on 171 seizure and 171 non-seizure segments. Each seizure segment contained, on average, 40 s of seizure data.

## 4. Description of EEG Dataset

The dataset was collected from the online Children’s Hospital Boston-Massachusetts Institute of Technology (CHB-MIT) and Khyber Hospital, Srinagar, Kashmir, for cross-verification. The experiments were done independently on both datasets. The dataset from CHB-MIT contained EEG recordings of 23 juvenile patients and 12 juvenile patients from Khyber Hospital, Srinagar, over 23 channels using 21 electrodes on the internationally recognized 10–20 electrode positioning system. The EEG signals were sampled at 256 Hz and filtered using 0 and 128 Hz bandpass filters. We trained our model on EEG segments of lengths 1, 2, and 4 s without overlapping. EEG segments are represented as an L * N matrix, where *L* represents the sequence length and *N* is the number of channels. We evaluated our models with 6000 data instances for 1-s normalized data, 3000 instances for 2-s length data, and 1500 instances for 4-s length data. The ictal and interictal segments were put in a matrix defined by the J * M matrix, where *J* is the length of the sequence and *M* is the number of channels. For cross-verification, the dataset from Khyber Hospital consisted of EEG recordings of 12 patients of 23.6-s duration. It was, in a similar way, recorded over 23 channels using 21 electrodes on the 10–20 electrode positioning system. Similarly, we trained our model on EEG segments of length 1, 2, and 4 s without overlapping. The data instances after normalization were recorded as 4000 for 1 s, 2000 for 2 s, and 1000 for 4 s, respectively. EEG segments were represented as an L * N matrix, where L represents the sequence length and *N* is the number of channels. The ictal and interictal segments were put in a matrix defined by the J * M matrix, where *J* is the length of the sequence and *M* is the number of channels. Patients with epilepsy typically have fewer seizures across substantially shorter spans than seizure-free intervals. Patients with epilepsy typically experience fewer episodes, which last for shorter lengths, than seizure-free intervals. Sometimes, interictal and ictal EEG data segment counts are different. When creating the final dataset, the number of interictal segments was chosen to be equal to the number of ictal segments to overcome the bias in the training process of the classification models, in which classifiers tend to favor the class with the biggest number of segments. It causes severe seizure detection performance in both favorable and adverse circumstances.

## 5. Methodology

**Figure 2:**
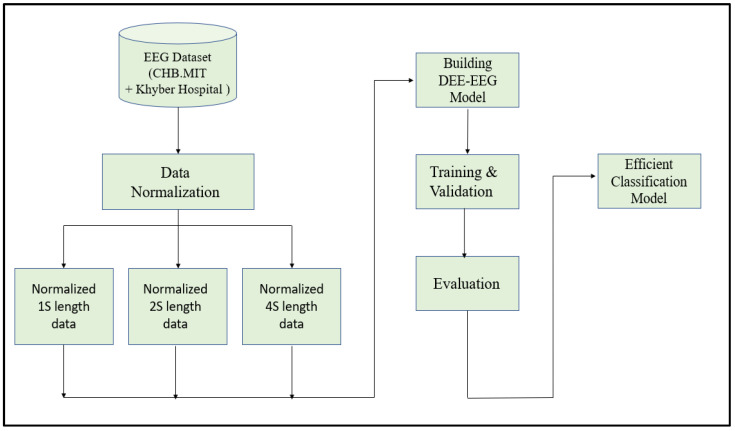
The flow diagram of the adopted methodology.

**Figure 3:**
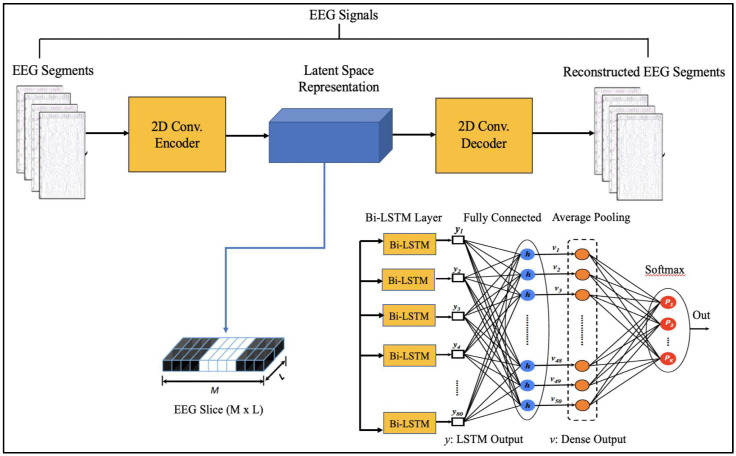
Proposed Architecture.

Since EEG signals are time series sequential data, recurrent neural networks like Long Short-Term Memory have been used to model architectures for classifying EEG signals into a seizure and non-seizure types. We adopted a deep learning approach, the flow diagram of which is shown in Figure 2. The data collected from CHB-MIT and Khyber Hospital were preprocessed slightly by normalizing them after dividing the EEG recordings into segments of 1 s, 2 s, and 4 s. Each one-dimensional EEG signal of size d was reshaped into a two-dimensional slice of size (M * L), where M is the number of time steps and *L* is the EEG segment length, as shown in Figure 3. The data segments were fed to deeply built models for training and evaluation. The models were evaluated on different performance matrices, and an optimized model was chosen as the best-performing model. Prediction is an essential and challenging part of time series data analysis. Seasonality, unexpected events, and internal changes, which also add to the data, affect prediction, accuracy, and efficiency. Epileptiform patterns require careful consideration because EEG is unique in its ability to support a clinical diagnosis of epilepsy. Certain benign patterns may be epileptiform, but they can occur in healthy people who do not have epilepsy. Understanding normal EEG and benign variants will aid in reducing over-interpretation and potentially avoiding overtreatment of patients during routine clinical practice. For the best performance, we employed an encoder-decoder-based Neural Network where input information is compressed to low dimensions by the encoder and decompressed to reconstruct the original signal, accomplished by continuously training the network by minimizing the loss function. In the Encoder-Decoder architecture, the input sequence is read in its entirety and encoded to a fixed-length representation. Then, a decoder uses this representation to output sequences until the end of the sequence is reached. Bi-LSTMs are used for both encoding and decoding. We have named our model the DCAE-ESD-Bi-LSTM model. This model can automatically learn the signal features from the labeled input data using supervised learning. Figure 3 shows the architecture of the proposed model. A brief description of Bi-LSTM and its working is given in the next section.

### 5.1. Dataset Preparation

The EEG dataset was preprocessed to guarantee that all values were standardized by having a zero mean (μ) and unit standard deviation (σ) using Equation (1). This was done by combining all the segments and applying z-score normalization for all the channels.

(1) $$x=\frac{x-\mu }{\sigma }$$

To ensure that the values in the original and the reconstructed segments have the same range, the values in the entire dataset were batch-scaled to the [0, 1] range using Min-Max normalization.

### 5.2. Proposed Architecture

The suggested models incorporate robust features that are automatically learned and contribute to the excellent classification accuracy of minimally preprocessed EEG signals. Our goal is to replace the burdensome manual feature extraction procedure and sophisticated systems that take a long time to train with a much more straightforward, quick, and effective method that takes advantage of AEs’ structure and capability. An encoder and a decoder are the two subnetworks that make up an AE neural network. The decoder is employed in a reverse manner to decompress or rebuild the original signal after the encoder network has compressed (encoded) the input information (EEG signals in our example) into a lower-dimensional representation. The CNN AE encoder subnetwork alternates four convolutional and four max-pooling layers. Max-pooling layers down sample dimensionality while convolutional layers learn spatial and temporal features in input EEG signal segments. A single convolutional layer has filters (kernels) with trainable parameters (weights) that slide over and convolve with input to generate feature maps with the same number of feature maps as filters. Stride controls filter window movement across the input. Downsampling simplifies pooling layer computation. Low-dimensional encoding network output is latent space representation or bottleneck. Four interchangeable convolutional and up-sampling layers reassemble the input in the decoder subnetwork. All encoder network convolutional layers have 32, 32, 64, and 64 filters. The decoder network’s first three convolutional layers have 64, 32, and 32 filters, while the last has one. By repeatedly training a network to rebuild its input while attempting to minimize the loss function between the original input and the reconstructed one, AE-based compression is carried out. We present 2D-DCAE-based models for supervised training to automatically learn inherent signal properties from labeled EEG segments. The latent space representation of the EEG signals is passed to different neural networks like Multilayer perceptron (MLP), LSTM, and finally, the proposed Bi-LSTM network, as shown in Figure 3.

### 5.3. Bidirectional Long Short-Term Memory

Bidirectional Long Short-Term Memory is inspired from Bi directional Recurrent Neural Networks that process sequential data, such as time series, textual data, etc., in both forward and backward directions by employing two hidden layers [37]. LSTM stores the past information since it reads the input data in the forward direction only, while in Bi-LSTM, the inputs are processed in parallel ways, one from past to future (forward move) and the other from future to past (backward move). The output from these two moves is merged to produce the final output. LSTMs send more contextualized, crucial training information via “cell states” than RNNs. The gated cell architecture saves key information obtained earlier in the time step sequence, allowing the model to make more educated predictions based on larger time step collections without losing context. “Bidirectionality” allows the LSTM to learn forward and backward input sequences, concatenating and embedding both interpretations in hidden states (in this demonstration, added as a wrapper to the first hidden layer of the model) [38,39]. The bidirectional LSTM network saves future information in reverse, providing context for prediction [40,41].

### 5.4. Network Configuration

A deep learning model for epilepsy seizure detection, ESD-Bi- LSTM, differentiates between epileptic and non-epileptic EEG. The proposed model non-linearly transforms the EEG segments into feature vectors automatically on very minimally preprocessed data, thus eliminating the overhead induced by the manual feature extraction methods. Our DCAE- ESD-Bi LSTM is trained by optimizing the cross-entropy cost function with the ‘adam” optimizer. The total number of Bi LSTM cells was set to 80 nodes in each hidden layer with a batch size of 50 and a fully connected dense layer set to 50 nodes. The batch size was set to 50, and the network converged after 1800 iterations with 40 epochs. The data was augmented by downsampling. The implementation was done in python using Keras and TensorFlow. Some of the previous models in the literature were also implemented for cross-validation and our method performed more accurately.

### 5.5. Performance Metrics for Evaluation

The commonly used metrics like accuracy, sensitivity, specificity, precision, and F1 score were used to evaluate the model performance [42]. These metrics were calculated to assess the classification against the test set using 10-fold cross-validation [1]. These performance metrics are defined as follows.

1. Precision (predicted positive value): It is the ratio of total samples which are epileptic and are correctly classified as epileptic (true positive) to the total number of data instances, which is the sum of those correctly classified as epileptic (true positive) and falsely classified as epileptic (false positive). It is given by:
$$precision(p)=\frac{TP}{TP+FP}$$
2. Recall: It is also termed as the sensitivity and is expressed as the ratio of correctly predicted positive, i.e., (epileptic correctly classified as epileptic) and the sum of total instances correctly classified as positive (true positive) and instances correctly classified as negative (true negative).
$$recall(r)=\frac{TP}{TP+FN}$$
3. F1 score: Recall and precision are transformed into another metric called the F1-score, which represents a harmonic mean of both. The F1 score combines the values of precision and recall in a single metric. It is given by:
$$F1-score=\frac{2*TP}{2*TP+FP+FN}$$
4. Accuracy: It is the ratio of correctly predicted (true positive and true negative) examples to the total number of examples. It is given by:
$$accuracy=\frac{Number\;of\;correct\;predictions}{Total\;number\;of\;predictions}$$
  For binary classification, it is denoted as:
  $$accuracy=\frac{TP+TN}{TP+TN+FP+FN}$$
5. Specificity: It is the ratio between true negative (TN) and the sum of true negative (TN) and false positive (FP). It determines the ability of the model to estimate healthy cases correctly. It is given by:

$$specificity=\frac{TN}{TN+FP}$$

## 6. Results and Discussion

We developed four models, namely DCAE + MLP, DCAE + LSTM, DCNN + MLP, and DCAE + ESD-Bi-LSTM. For 10-fold cross-validation, we observed the performance of the implemented models on five metrics. The five metrics are accuracy, precision, specificity, sensitivity, and F1 score. All four models were fed with data of different lengths. The lengths were 1 s, 2 s, and 4 s. The confusion matrix for 4s length EEG segments is given in Table 1. The average of all performance metrics over the 10-fold evaluation method is given in Table 2. In all the models mentioned above, a dropout of 0.75 was applied to the hidden layers to avoid overfitting. The loss of the model has been calculated using the log loss method. In the fully connected dense layer, the SoftMax activation function was used to perform the classification work. To visually interpret the results, the following graphs were plotted. In the first instance, a Deep Convolutional neural network was coupled with a Multilayer Perceptron model on the normalized EEG lengths of 1 s, 2 s, and 4 s. The model produced an accuracy of 96.2% on 2-s EEG length, 97.7% accuracy on 2-s EEG length, and 98.1% accuracy on 4-s length EEG. The same data produced accuracies of 97.3%, 98.5%, and 98.5% on the model, based on the Deep Convolutional Autoencoder model and coupled with Multilayer Perceptron, on EEG lengths of 1 s, 2 s, and 4 s, respectively.

**Figure 4:**
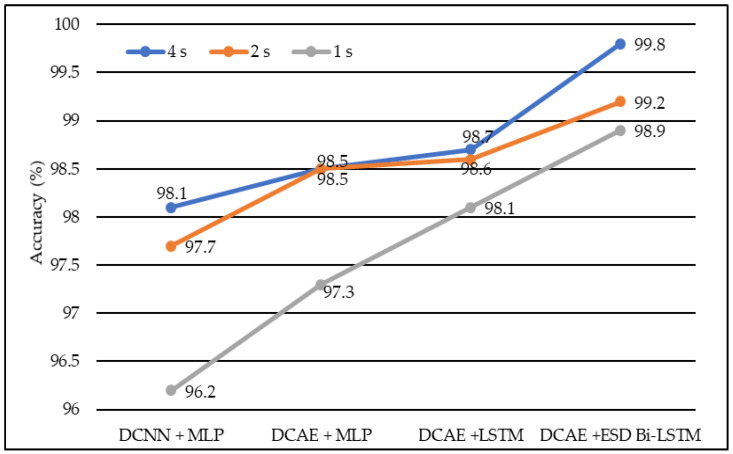
The average accuracy of different models using different EEG segment lengths.

**Figure 5:**
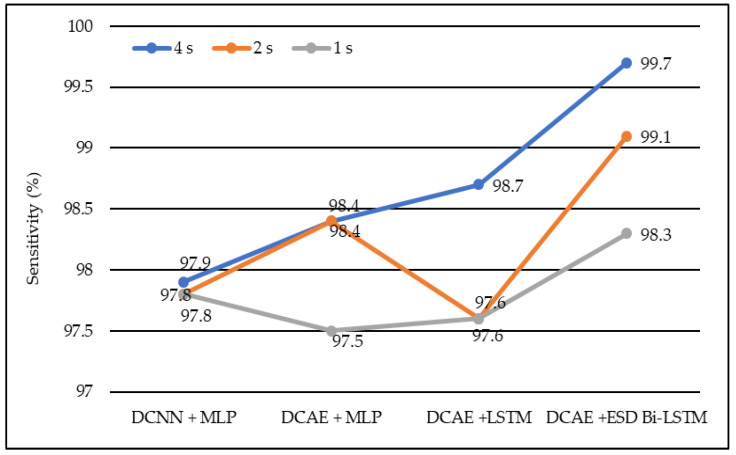
Average sensitivity of different models using different EEG segment lengths.

Similarly, Deep Convolutional Neural Network coupled with Long Short-Term Memory (LSTM) produced accuracies of 98.1%, 98.6%, and 98.7% on EEG lengths of 1 s, 2 s, and 4 s, respectively as shown in Figure 4. Finally, our proposed model, based on the Deep Convolutional Autoencoder model and Epileptic Seizure Detection-Bidirectional-Long Short-Term Memory, outperformed other implemented models, producing an accuracy of 98.9%, 99.2% and 99.8% on the EEG segment lengths of 1 s, 2 s, and 4 s, respectively. The confusion matrix for the 4-s length data representation with a total count of 2500 data instances is described in Table 1. Figure 5 represents the accuracy of different models on different EEG lengths.

In the same manner, DCNN + MLP produced sensitivities of 97.8%, 97.8%, and 97.9% on 1-s, 2-s, and 4-s EEG segment lengths, respectively. DCAE + MLP produced sensitivities of 97.5%, 98.4%, and 98.4% on EEG segment lengths of 1 s, 2 s, and 4 s, respectively. DCAE + LSTM produced 97.6%, 97.8%, and 98.7% sensitivities on 1-s, 2-s, and 4-s EEG segment lengths, respectively. The proposed DCAE + ESD-Bi-LSTM produced 98.3%, 99.1%, and 99.7% sensitivities on 1-s, 2-s, and 4-s EEG segment lengths, respectively, which are the best among the implemented models, as shown in Figure 5.

**Figure 6:**
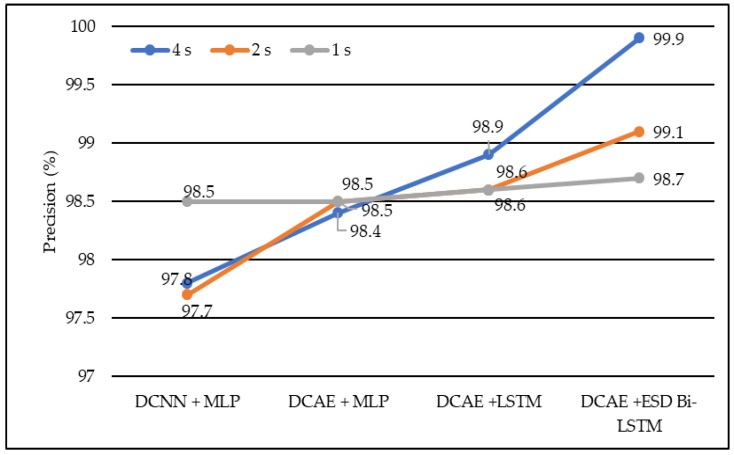
Average precision of different models using different EEG segment lengths.

In terms of precision, DCNN + MLP produced precision of 97.7%, 97.8%, and 98.5% on 1-s, 2-s, and 4-s EEG segment lengths, respectively. DCAE + MLP produced precision of 98.4%, 98.5%, and 98.9% on EEG segment lengths of 1 s, 2 s, and 4 s, respectively. DCAE + LSTM produced 98.6%, 98.6%, and 98.9% precision on 1-s, 2-s, and 4-s EEG segment lengths, respectively. The proposed DCAE + ESD-Bi-LSTM produced 98.7%, 99.1%and 99.9% precision on 1-s, 2-s, and 4-s EEG segment lengths, respectively, which are the best among the implemented models, as shown in Figure 6.

**Figure 7:**
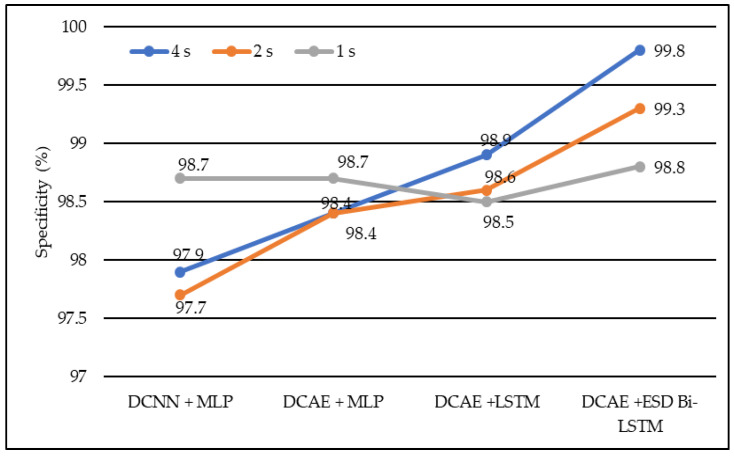
Average specificity of different models using different EEG segment lengths.

In terms of specificity, DCNN + MLP produced specificities of 97.7%, 97.9%, and 98.7% on 1-s, 2-s, and 4-s EEG segment lengths, respectively. DCAE + MLP produced specificities of 98.4%, 98.4%, and 98.7% on EEG segment lengths of 1 s, 2 s, and 4 s, respectively. DCAE + LSTM produced 98.5%, 98.6%, and 98.9% specificities on 1-s, 2-s, and 4-s EEG segment lengths, respectively. The proposed DCAE + ESD-Bi-LSTM produced 98.8%, 99.3%, and 99.8% specificities on 1-s, 2-s, and 4-s EEG segment lengths, respectively, which are the best among the implemented models, as shown in Figure 7.

**Figure 8:**
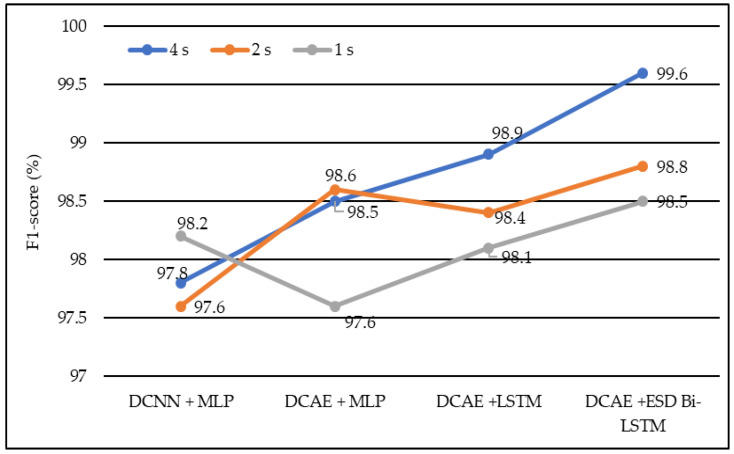
Average F1-score of different models using different EEG segment lengths.

In terms of the F1 score, DCNN + MLP produced F1 scores of 97.6%, 97.8%, and 98.2% on 1-s, 2-s, and 4-s EEG segment lengths, respectively. DCAE + MLP produced F1 scores of 97.6%, 98.5%, and 98.6% on EEG segment lengths of 1 s, 2 s, and 4 s, respectively. DCAE + LSTM produced 98.1%, 98.4%, and 98.9% F1 Scores on 1-s, 2-s, and 4-s EEG segment lengths, respectively. The proposed DCAE + ESD-Bi-LSTM produced 98.5%, 98.8%, and 99.6% F1 Scores on 1-s, 2-s, and 4-s EEG segment lengths, respectively, which are the best among the implemented models, as shown in Figure 8.

The models shown in Table 2 were individually executed, and the values of the various performance metrics were evaluated for each of the models. The DCAE-ESD-Bi-LSTM model had 80 nodes in the hidden layer with a batch size of 10, followed by the fully connected layer having 50 nodes. The average pooling layer follows the fully connected layer. The output of the pooling layer is fed to the SoftMax layer, which provides a binary output classifying the data into epileptic and non-epileptic instances. It was found that the proposed model was able to outperform all other methods using a smaller number of layers. The proposed model worked best when the EEG segment length was 4 s, with an average accuracy of 98.9%, an F1 score of 98.8%, a sensitivity of 98.3%, an F1 score of 98.7%, and an F1 score of 98.5% for 1-s EEG data segment length; an accuracy of 99.2%, F1 score of 99.3%, the sensitivity of 99.1%, F1 score of 99.1% and F1 score of 98.8% for 2-s EEG data segment length; an accuracy of 99.8%, F1 score of 99.8%, the sensitivity of 99.7%, F1 score of 99.9% and F1 score of 99.6% for 4-s EEG data segment length on 10-fold cross-validation system. The model worked best on the 4-s segment of EEG data. The proposed system outperformed all previous methods on all EEG segment lengths; a comparison of our proposed system using different EEG segment lengths is given in Figure 8.

### Loss Function and Optimization

**Figure 9:**
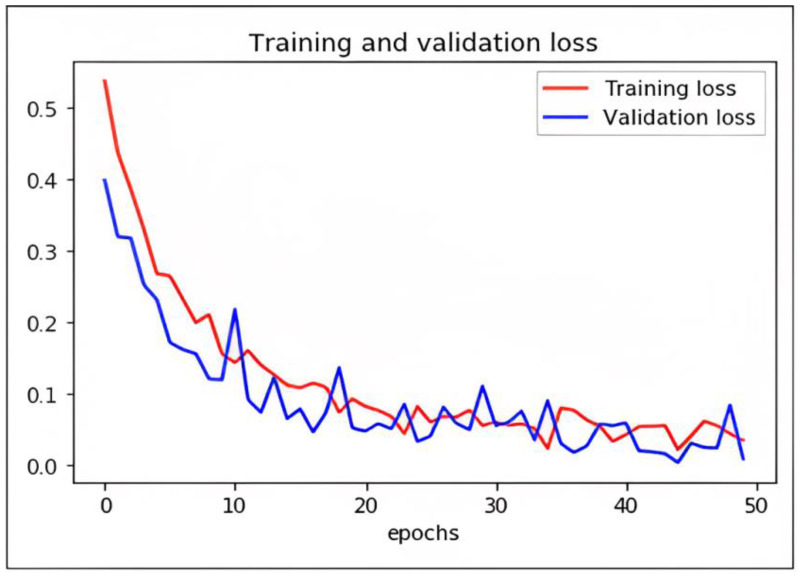
Graph plot representing loss with epochs of DCAE + ESD-Bi-LSTM.

While DCAE-ESD-Bi-LSTM carries out the two input tasks simultaneously, our proposed categorization and reconstruction model aims to reduce network traffic losses during training. We calculated the losses between the actual and projected class labels. Figure 9 shows a 2D plot of the number of epochs vs. validation and training loss to show how the proposed model works. When differentiating precisely, the log loss graph is easily comprehended and provides an accurate view of evaluating the model performance. Higher accuracy scores and lower loss values show that the model successfully performs the categorization task. The outcomes of our tests indicate that this model is much superior to every other model that has lately been developed in this field.

We have trained the network using different optimizers. Based on the performance of the optimizers like ADADELTA, SGD, and RMS, we found ADAM optimizer produced the best results with a learning rate of 0.0001. The loss in the proposed model is shown as follows.

## 7. Comparison with Other Methods

In the literature, many models have been used for detecting seizures using different algorithms and evaluated using different parameters. All previous authors did not use the same metrics for evaluating their models. Therefore, we compared our results on the most commonly used parameters, i.e., accuracy, sensitivity, and F1 score. The comparisons are given in Table 3. Seizure states have been identified with an accuracy, sensitivity, and specificity of 98%, 98%, and 97%, respectively, by Ke et al. [43]. They applied VGGNET on the same dataset. Aarabi et al. [44] applied BNN and achieved an accuracy of 93%, an F1 score of 95%, and a sensitivity of 91%. In a similar case, Subasi et al. [45] applied the ME classifier and achieved an accuracy of 94.5%, an F1 score of 94%, and a sensitivity of 95%. Chandaka et al. [46] proposed a model using an SVM classifier and obtained an accuracy of 95.96%, an F1 score of 93%, and a sensitivity of 92%. Yaun et al. [47], applied an ELM classifier and obtained an accuracy of 96.5%, an F1 score of 96%, and a sensitivity of 92.5%. Yaun et al. [48] applied a single-layer feed-forward network SLFN and produced an accuracy of 96.5%. Hossain [49] applied deep CNN and obtained an overall sensitivity of 90.00%, specificity of 91.65%, and accuracy of 98.05% for 23 patient cross-patient EEG data. The comparison shows that our proposed model is the best-performing model, with the best accuracy rate of 99.9%, F1 score of 99.7%, and sensitivity of 99.8% on the 4-s EEG segment length. Figure 9 shows the comparative results. The comparison shows that our proposed model is the best-performing model with the best accuracy rate of 99.9%, F1 score of 99.7%, and sensitivity of 99.8%.

## 8. Conclusions

In this work, a deep learning method for the autonomous detection of seizures using EEG signals has been proposed, namely the DCAE-ESD-Bi-LSTM, which has outperformed the previous methods from the literature. Compared to the fundamental techniques, this strategy can pick up high-level EEG representations and effectively differentiate between normal and EEG activity during seizures. Another benefit of this strategy lies in its resistance to typical EEG artifacts (such as muscle white noise is included as well as eye movement and activity). The suggested technique has been evaluated and contrasted using the UHB MIT and dataset from Khyber Hospital, India, to several cutting-edge techniques. The results demonstrate the superiority and effectiveness of the suggested approach in identifying epileptic seizures. It produces strong seizure detection in both good and bad situations.

## Figures and Tables

**Table 1:** Confusion matrix.

		Predicted	
		Negative(N)	Positive(P)
Actual	Negative	723	1
	Positive	4	1772

**Table 2:** Performance metrics of different models with different EEG segment lengths.

	Model	Accuracy	Sensitivity	Precision	Specificity	F1 Score
1 s	DCAE + MLP	97.3	97.5	98.7	98.5	97.6
	DCAE + LSTM	98.1	97.6	98.5	98.6	98.1
	DCNN + MLP	96.2	97.8	98.7	98.5	98.2
	DCAE + ESD Bi-LSTM	98.9	98.3	98.8	98.7	98.5
2 s	DCAE + MLP	98.5	98.4	98.4	98.5	98.6
	DCAE + LSTM	98.6	97.6	98.6	98.6	98.4
	DCNN + MLP	97.7	97.8	97.7	97.7	97.6
	DCAE + ESD Bi-LSTM	99.2	99.1	99.3	99.1	98.8
4 s	DCAE + MLP	98.5	98.4	98.4	98.4	98.5
	DCAE + LSTM	98.7	98.7	98.9	98.9	98.9
	DCNN + MLP	98.1	97.9	97.9	97.8	97.8
	DCAE + ESD Bi-LSTM	99.8	99.7	99.8	99.9	99.6

**Table 3:** Comparison of proposed DCAE-ESD-Bi-LSTM with other state-of-the-art models.

Method	Year	Classifier	Sensitivity (%)	F1 Score (%)	Accuracy (%)
Ke et al., [43]	2018	MIC + VGGNET	98.1	NA	98.5
Aarabi et al., [44]	2006	BNN	91.00	95.00	93.00
Subasi, [45]	2007	ME	95.00	94.00	94.50
Chandaka et al., [46]	2009	SVM	92.00	93.00	95.96
Yuan et al., [47]	2011	ELM	92.50	96.00	96.50
Zhou et al., [48]	2018	SLFN	NA	NA	96.5
M. Shamim Hossain et al., [49]	2019	Deep CNN	95.65	91.65	90.00
Proposed method	2022	DCAE-ESD-Bi-STM	99.8	99.7	99.8
